# Characterization of Swallowing Sound: Preliminary Investigation of Normal Subjects

**DOI:** 10.1371/journal.pone.0168187

**Published:** 2016-12-13

**Authors:** Tsuyoshi Honda, Takuro Baba, Keiko Fujimoto, Takaharu Goto, Kan Nagao, Masafumi Harada, Eiichi Honda, Tetsuo Ichikawa

**Affiliations:** 1 Department of Oral and Maxillofacial Prosthodontics, Institute of Biomedical Sciences, Tokushima University Graduate School, Tokushima, Japan; 2 Department of Radiology, Institute of Biomedical Sciences, Tokushima University Graduate School, Tokushima, Japan; 3 Department of Oral and Maxillofacial Radiology, Institute of Biomedical Sciences, Tokushima University Graduate School, Tokushima, Japan; Northwestern University Feinberg School of Medicine, UNITED STATES

## Abstract

**Objective:**

The purpose of this study was to characterize the swallowing sound and identify the process of sound generation during swallowing in young healthy adults.

**Methods:**

Thirty-three healthy volunteers were enrolled and allocated into three experimental groups. In experiment 1, a microphone was attached to one of eight cervical sites in 20 subjects, participants swallowed 5 ml water, and the sound waveform was recorded. In experiment 2, 10 subjects swallowed either 0, 5, 10, or 15 ml water during audio recording. In addition, participants consumed the 5 ml bolus in two different cervical postures. In experiment 3, the sound waveform and videofluoroscopy were simultaneously recorded while the three participants consumed 5 ml iopamidol solution. The duration and peak intensity ratio of the waveform were analyzed in all experimental groups.

**Results:**

The acoustic analysis of the waveforms and videofluoroscopy suggested that the swallowing sound could be divided into three periods, each associated with a stage of the swallowing movement: the oral phase comprising posterior tongue and hyoid bone movement; the pharyngeal phase comprising larynx movement, hyoid bone elevation, epiglottis closure, and passage of the bolus through the esophagus orifice; and the repositioning phase comprising the return of the hyoid bone and larynx to their resting positions, and reopening of the epiglottis.

**Conclusion:**

Acoustic analysis of swallowing sounds and videofluoroscopy suggests that the swallowing sound could be divided into three periods associated with each process of the swallowing movement: the oral phase comprising the posterior movement of the tongue and hyoid bone; the pharyngeal phase comprising the laryngeal movement, hyoid bone elevation, epiglottis closure, and the bolus passage to the esophagus orifice; and the repositioning phase comprising the repositioning of the hyoid bone and larynx, and reopening of the epiglottis.

## Introduction

In general, many East Asian (particularly Japan) and European countries now face a severe problem with regard to aging population. An ultra-aged society is generally defined as a society with more than 22% of its total population comprising elderly people. In 2008, more than 22% of the Japanese population was comprised of the elderly, i.e., people aged 65 years or older; hence, making Japan an ultra-aged society.

Dysphagia is a symptom common to many diverse conditions in the elderly and causes serious degradation of activities of daily living [1]. Dysphagia increases the risk of aspiration pneumonia and malnutrition [2–5]. Therefore, it is important to evaluate the risk of dysphagia early. A variety of clinical bedside examinations have been proposed to screen for dysphagia [6–9]; the repetitive saliva swallowing test, modified water swallow test, and food test are among the commonly used methods in Japan. Cervical auscultation, during which swallowing sounds are listened to and assessed using a stethoscope, is a very simple, effective, and commonly utilized method [10–16]. This method is based on the assumption that the sound differs during abnormal swallowing. Dysphagia criteria for cervical auscultation are based on the following symptoms and signs: duration and magnitude of the sound; abnormal sound properties (i.e., bubbling sound); and coughing or respiration sounds immediately after swallowing. Hirano reported that the diagnostic concordance rate between cervical auscultation and videofluoroscopy was 83.5% [17]. Takahashi similarly reported a 77.3% rate using the sound duration and magnitude as the diagnostic criteria [18]. However, this technique is difficult to evaluate objectively, and it is unclear how sound is generated during swallowing [19]. The relationship between the swallowing sound and processes requires clarification to ensure that dysphagia can be adequately examined using cervical auscultation.

The aim of this study is to characterize the swallowing sound and identify the process of sound generation during swallowing in young healthy adults.

## Material and Methods

### Participants

Volunteer participants were recruited from undergraduate and graduate students at Tokushima University based on the following inclusion criteria: i) normal dentition, ii) no history of stomatognathic disorder or dysphagia, iii) repetitive saliva swallowing test (RSST) value greater than three [7,8], and iv) provision of informed consent prior to study enrolment. Thirty-three young, healthy volunteers were enrolled and allocated to three experimental groups as follows: Experiment 1: 10 men and 10 women, mean age 25.8 y; Experiment 2: five men and five women, mean age 28.0 y; and Experiment 3: three men, whose thyroid cartilage movements are easily detected for measurement and analyses, mean age 27.6 y. All relevant approvals were obtained from the clinical research ethical review board of Tokushima University Hospital (No. 1406), and this study was conducted in accordance with the ethical principles stated by the Declaration of Helsinki. All the participants signed a written informed consent before study participation

### Bolus

A commercially available natural spring water (CRYSTAL GEYSER, Otsuka Foods Company Ltd, Japan) at approximately 25°C was used in Experiments 1 and 2. Iopamidol solution (Iopamiron 300, Bayer Yakuhin, Ltd, Japan) diluted 1:3 with water was used in Experiment 3.

### Acoustic recording

**Fig 1:**
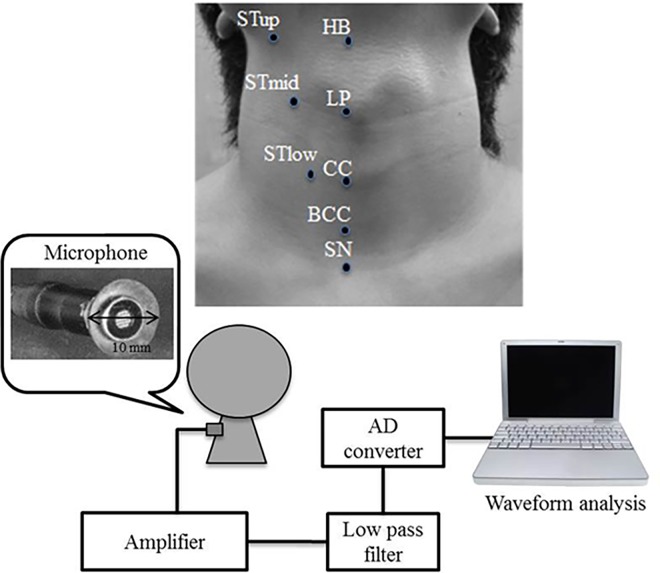
Swallowing sound recording system and eight cervical sites for microphone attached. (HB: center of the hyoid bone; LP: center of the laryngeal prominence; CC: center of the cricoid cartilage; BCC: center of the ventral cricoid cartilage; SN: suprasternal notch; STup: intersection of the lateral HB and the front edge of the sternocleidomastoid muscle; STmid: intersection of the lateral LP and the front edge of the sternocleidomastoid muscle; STlow: intersection of the lateral CC and the front edge of the sternocleidomastoid muscle).

The acoustic recording system is illustrated in Fig 1. An ultra-small condenser microphone (AT9903, Audio-Technica, Tokyo, Japan; frequency: 30–18000 Hz) was used to detect the swallowing sounds. The sensor tip was enlarged to a 10 mm diameter using an auto-curing resin (UNIFAST II Clear, GC, Tokyo, Japan) to ease its attachment to the skin. The microphone was attached to the cervical skin with double-sided tape (1517, 3M Japan, Tokyo, Japan). The output signal was increased with an amplifier (AT-MA2, Audio-Technica) and a low-pass filter (NF 3611, NF Co., Yokohama, Japan; frequency: 10 kHz, -24 dB ± 2 dB dB/oct). The signals were digitized at a 16 bit resolution and 20 kHz sampling rate using an analog-digital card (BPC-0600, Interface Co., Hiroshima, Japan), and analyzed with waveform analysis software (DADiSP, CAE Solutions Co., Tokyo, Japan) on a personal computer. The microphone was attached to one of eight cervical sites as follows: HB: center of the hyoid bone; LP: center of the laryngeal prominence; CC: center of the cricoid cartilage; BCC: center of the below cricoid cartilage; SN: suprasternal notch; STup: intersection of the lateral HB and the front edge of the sternocleidomastoid muscle; STmid: intersection of the lateral LP and the front edge of the sternocleidomastoid muscle; and STlow: intersection of the lateral CC and the front edge of the sternocleidomastoid muscle. The swallowing sounds in every site were recorded three times.

### Experiment 1: Influence of cervical location on swallowing sounds

Participants were seated in a soundproof room, on a straight-backed chair, and in an upright position. The microphone was attached to the skin at one of the eight sites around the neck (Fig 1). The order of the measurement sites was randomized across the subjects. Participants were instructed to consume 5 ml water (administered sublingually through a syringe) in one complete swallow with a normal chin position. The recording was started 2 s before and ended 2 s after the swallow maneuver. Measurements were repeated three times at a 3-min interval to recover oral wetness and eliminate the influence of last swallowing. It took about one hour and half to complete the serial experiment in every patient

### Experiment 2: Influence of bolus volume and cervical posture on swallowing sounds

Acoustic recording was performed as in Experiment 1. The microphone was attached onto the skin over the laryngeal prominence (LP) where the three peaks were clearly observed and the site was specified clearly as an anatomical landmark. Four oral materials were prepared as follows: 0 (dry swallow), 5, 10, and 15 ml water. During the 5 ml swallow, participants performed the maneuver in a normal chin position and chin-down position. The measurement order in the 5 conditions was randomly done and the measurements were repeated three times in every condition at a 3 min interval. It took about 45 min to complete the serial experiment in every patient.

### Experiment 3: Simultaneous acoustic recording and videofluoroscopy

Participants were seated in the radiography room, on a straight-backed chair, and in an upright position. The microphone was attached to the LP site. Participants were instructed to consume 5 ml iopamidol solution water as in Experiment 1, and swallowing was simultaneously recorded fluoroscopically (Ultimax FPD, TOSHIBA MEDICAL SYSTEMS CO, Japan; tube voltage: 69 kV, tube current: 1.0 mA). The acoustic and visual signals were transferred to a visual recorder (AQ-VU, TEAC, Tokyo, Japan). The measurements were repeated three times at a 3-min interval. The visual signals were recorded at 30 frames per second and the acoustic signals at a 16 bit resolution and 2 kHz sampling rate. The images were qualitatively inspected while the synchronizing window was set to the swallowing sound signal in the monitor by multiple examiners.

### Acoustic analysis

**Fig 2:**
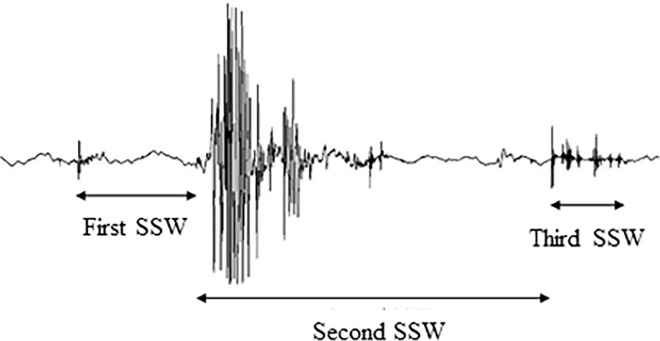
Representative swallowing sound waveform divided into three sections (recording site: LP).

A representative swallowing sound waveform for 5 ml water is shown in Fig 2. Each waveform was divided into three sections as described previously [20] and detailed below. The first swallowing sound wave (SSW) was defined as the period from the beginning of the first peak of the wave to the beginning of the largest subsequent peak, the second SSW as the period from the largest peak to the beginning of the reraising small peak of third SSW through continuous large peaks and silent periods, and the third SSW as the period from the reraising small peak over noise level to the end of the swallowing wave. Each waveform was segmented by one examiner using the noise level during the resting period as a reference. The duration and peak intensity ratio of each SSW were measured in Experiments 1 and 2. The peak intensity ratio was defined as the ratio between the peak-to-peak (P-P) value and the resting noise level before swallowing.

### Statistical analysis

Statistical analysis was conducted using SPSS® version 19.0 (IBM Corp., Armonk, NY, USA). Significance was designated at p < 0.05. The sample size, effect size, alpha level of 0.05, and statistical power of 0.8 were determined to be acceptable for the number of experimental groups. In Experiment 1, ANOVA with Bonferroni post-hoc test was used to compare the acoustic data between the eight cervical sites. For the multiple comparisons in Experiment 1, significance level was determined after Bonferroni adjustment. In Experiment 2, Spearman’s rank correlation coefficient was calculated to examine the relationship between the acoustic changes during swallowing and the volume of swallowed material. The Wilcoxon signed-rank test was used to examine the association between the acoustic changes and cervical posture.

## Results

### Experiment 1

**Fig 3:**
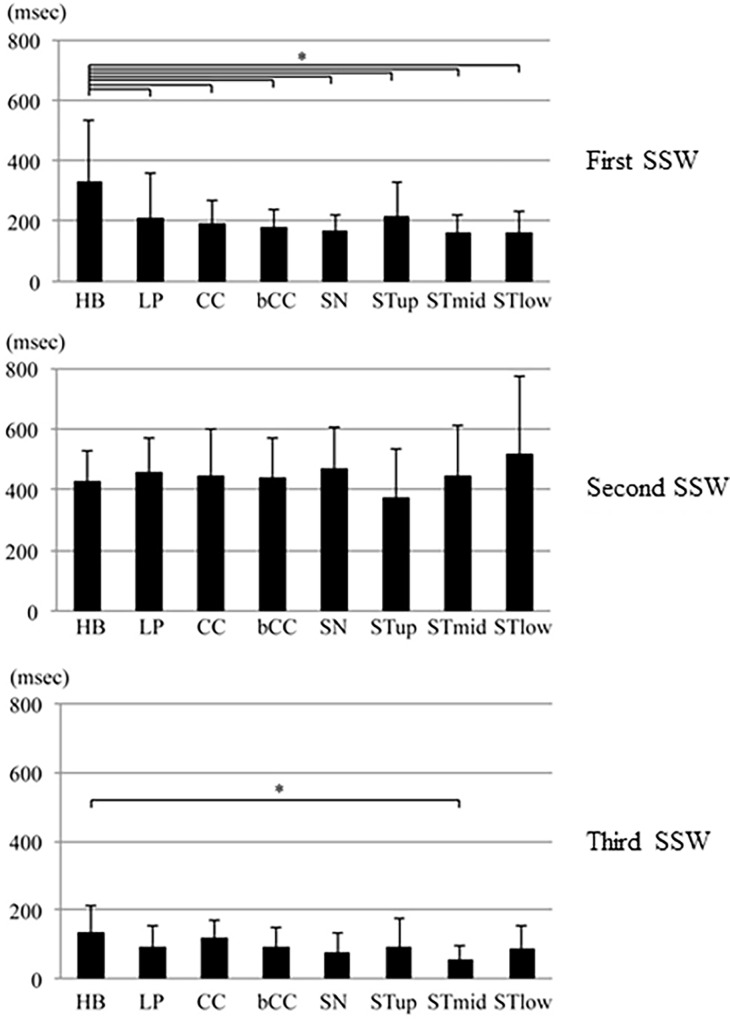
Duration (mean and standard deviation) of the first, second, and third swallowing sound waves at the eight cervical sites (*p<0.0018).

**Fig 4:**
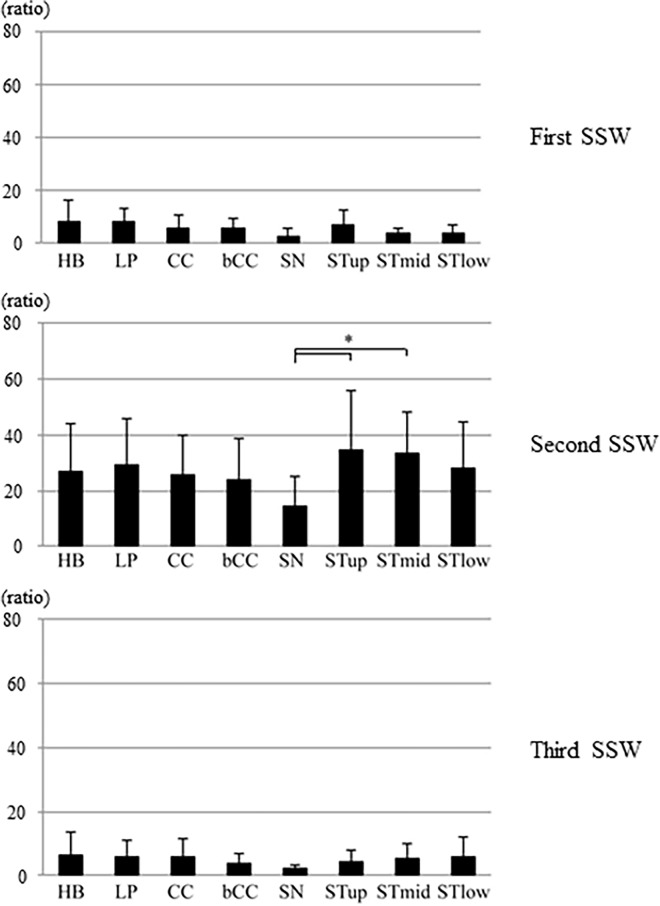
Peak intensity ratio (mean and standard deviation) of the first, second, and third swallowing sound waves at the eight cervical sites (*p<0.0018).

The means and standard deviations of duration and peak intensity ratio for each SSW at each recording site are shown in Figs 3 and 4. The means and standard deviations of duration of the entire waveform, first SSW, second SSW, and third SSW was 732 ± 201 ms, 203 ± 122 ms, 444 ± 159 ms, and 93 ± 68 ms, respectively. In the first SSW, The means and standard deviations of duration at the hyoid bone (HB) was 330 ± 200 ms, which was significantly longer than the mean durations at the other sites. The means and standard deviations of peak intensity ratios at the hyoid bone (HB) and STup sites were 8.0 ± 8.3and 6.8 ±5.8, respectively, which were higher than the ratios at the remaining sites. Both acoustic parameters during the second SSW were larger than the parameters during the other periods. There was no significant difference in the mean duration between the sites, but the duration at STlow was longest. The peak intensity ratios at the STup and STmid were significantly higher than the ratio at SN. The peak intensity ratios at lateral sites (STup, STmid, and STlow) were generally larger than the ratios at central sites (HB, LP, CC, BCC, and SN). Both acoustic parameters were less during the third SSW than during other periods. The mean duration was longest at the hyoid bone (HB), which was significantly longer than at STmid. No significant differences were observed between the hyoid bone and remaining sites. No significant difference was observed in the peak intensity ratio among the sites. Table 1 shows the standard deviations in intra-subject and inter-subject on peak intensity ratio and duration of each SSW. The standard deviations in intra-subject and inter-subject refer to the mean of SDs in 3 repetitions in intra-subject and the mean of SDs in the representatives of 20 subjects, respectively. The variation was low in intra-subjects compared to that in inter-subjects. No significant difference of the above wave characterizations was found between genders.

**Table 1:** The mean of intra and inter-subject standard deviations of the first, second, and third swallowing sound waves.

**Duration**	**HB**			**LP**			**CC**			**bCC**		
**First SSW**	119.7	/	179.9	68.3	/	145.1	42.2	/	75.2	43.2	/	63.3
**Second SSW**	81.7	/	166.3	92.2	/	171.7	86.1	/	178.4	73.8	/	151.5
**Third SSW**	41.3	/	72.5	28.5	/	55.7	27.2	/	56.6	27.4	/	47.9
**Duration**	**SN**			**Stup**			**Stmid**			**Stlow**		
**First SSW**	55.8	/	51.9	44.0	/	115.2	45.9	/	61.4	31.1	/	75.1
**Second SSW**	66.2	/	147.7	48.5	/	161.2	58.4	/	187.0	86.5	/	260.9
**Third SSW**	36.7	/	55.8	28.5	/	87.9	14.8	/	48.0	23.5	/	63.5
**Peak intensity ratio**	**HB**			**LP**			**CC**			**bCC**		
**First SSW**	4.0	/	8.4	3.3	/	5.4	2.3	/	5.0	2.2	/	3.8
**Second SSW**	11.5	/	17.5	10.7	/	16.7	9.4	/	13.0	8.8	/	15.1
**Third SSW**	4.1	/	7.8	2.5	/	5.5	2.6	/	5.4	1.5	/	3.0
**Peak intensity ratio**	**SN**			**Stup**			**Stmid**			**Stlow**		
**First SSW**	1.1	/	2.6	2.8	/	5.6	1.3	/	2.0	2.0	/	3.2
**Second SSW**	5.3	/	9.4	13.3	/	21.4	16.5	/	14.5	12.5	/	15.7
**Third SSW**	0.8	/	1.5	2.9	/	3.7	2.3	/	4.9	2.0	/	6.4

### Experiment 2

**Fig 5:**
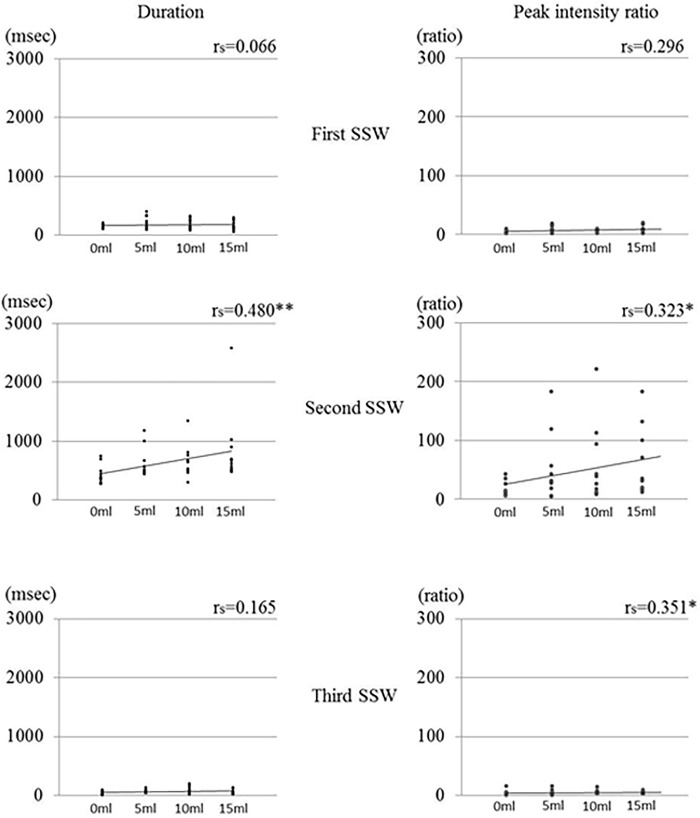
Duration and peak intensity ratio of the first, second, and third swallowing sound waves according to bolus volume (*p<0.05, **p<0.01).

Fig 5 shows the mean duration and peak intensity ratio of the first, second, and third SSWs for the various bolus volumes. No significant correlation was observed in the duration and peak intensity ratio during the first SSW for any of the bolus volumes. For the second SSW, a significant correlation was found between the bolus volume and the duration (p < 0.01) and peak intensity ratio (p < 0.05). During the third SSW, there was no significant correlation between the duration and bolus volume, but a significant correlation was found between the peak intensity ratio and bolus volume (p < 0.05).

**Fig 6:**
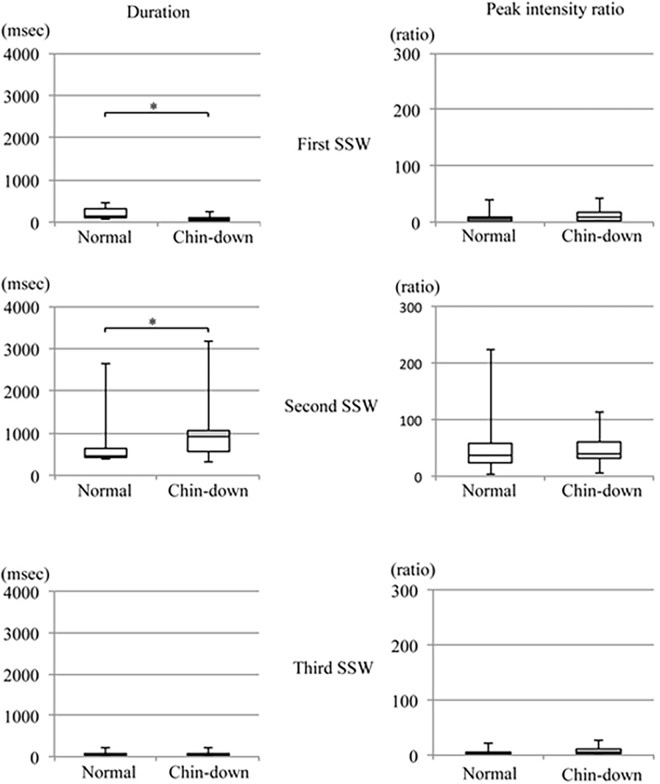
Duration and peak intensity ratio of the first, second, and third swallowing sound waves in normal and chin-down positions(*p<0.05).

Fig 6 shows the means of duration and peak intensity ratio during each SSW in normal and chin-down positions. The mean duration of the first SSW was significantly shorter in a chin-down position than in a normal chin position. The mean duration of the second SSW was significantly longer in a chin-down position than in a normal chin position. There was no significant difference in the mean duration of the third SSW between the two positions. There was no significant difference in the peak intensity ratio between the two postures during any SSW period.

### Experiment 3

**Fig 7:**
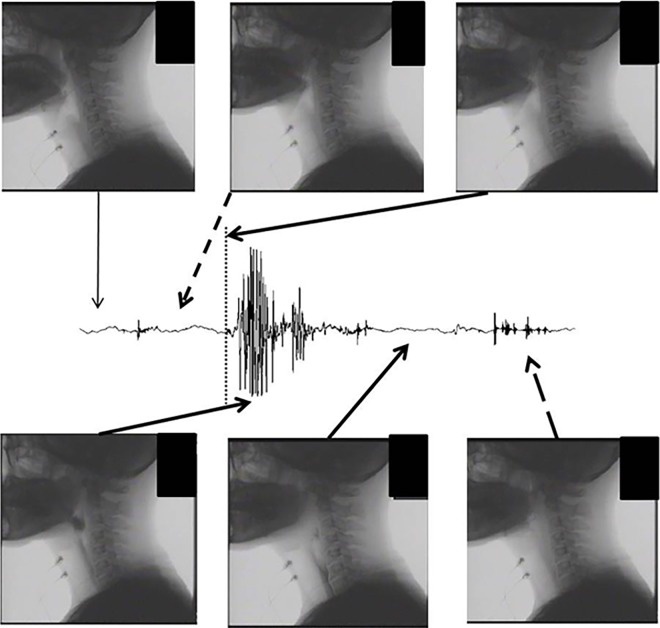
Representative profiles of recording simultaneously of swallowing sounds and videofluoroscopy (recording site: LP).

Fig 7 shows a representative videofluoroscopy for each SSW period. The oral stage, defined as the transfer of the bolus from the oral cavity to the pharynx with posterosuperior movement of the hyoid bone, occurred during the first SSW, especially around the peak. Tongue made a contact to the palate at the beginning of the first SSW and moved posterior at the peak. The food bolus passed through the posterior region of tongue by the beginning of the second SSW. The hyoid bone moved anterosuperior approximately at the beginning of the second SSW. The pharyngeal reflex, defined as larynx elevation, arrival of the bolus at the valleculae, and transfer from the pharynx to the esophagus through the esophageal orifice, occurred during the large peaks in the second SSW. The transfer of the bolus from the pharynx to the esophagus continued through the silent period of the second SSW. At the beginning of the third SSW, the bolus completely entered the esophagus; and the hyoid bone and larynx resumed their baseline positions, and the epiglottis reopened at the end of the third SSW. Similar findings were observed in all three participants.

## Discussion

In a previous study, we performed ultrasonography of the thyroid cartilage and thyroid gland positions [20]. Based on these previous findings, we speculated that swallowing sound generation could be assessed using the sound waveform recorded over the lateral laryngeal prominence. Our previous findings suggested that the swallowing waveform could be divided into three periods, each associated with one component of the swallowing movement: the oral phase, comprising tongue and hyoid bone movement; the pharyngeal phase, comprising larynx movement and passage of the bolus from the pharynx to the esophagus; and the repositioning phase, comprising return of the hyoid bone, larynx, and epiglottis to their resting positions. In this study, the swallowing sound waveforms at the eight sites were also divided into three major sections. Generally, the closer the sound source, the larger the sound pressure, and the earlier the sound is detected; therefore, we attempted to identify the source of sound events during the three periods considering this characteristic. In the first SSW, there was no significant difference in the peak intensity ratio between the cervical sites, but the peak intensity ratio was highest at the hyoid bone (HB), indicating that the first SSW was generated from the center of the hyoid bone. The duration of the first SSW was significantly longer at the hyoid bone than at the other sites. This suggests that the sound event during the first SSW was close to the hyoid bone, while the sound event during the second SSW was relatively far from the hyoid bone. This speculation is also supported by the videofluoroscopy results; during this period, the bolus was held by the tongue and transferred from the oral cavity to the pharynx.

During the second SSW, the peak intensity ratios were higher at the STup and STmid sites than at the other sites. Therefore, we surmised that the sound event during the second SSW was generated from the front edge of the sternocleidomastoid muscle. In addition, the duration was longer at the STlow than at the other sites, suggesting that the sound event during the second SSW was close to the STlow, while the sound event during the third SSW was relatively far from the STlow. By contrast, the duration during the second SSW was shortest at the STup, suggesting that the sound events during the second and third SSWs were generated near the STup. Based on the acoustic results from the first and second SSWs, the sound event during the third SSW was generated near the STup and far from the STlow. The mean peak intensity ratio during the third SSW was highest at the hyoid bone. Thus, the third SSW was likely generated near the hyoid bone, which is supported by the acoustic results of the first and second SSWs, and the videofluoroscopy results. During videofluoroscopy, the bolus completely entered the esophagus, the hyoid bone and larynx were repositioned, and the epiglottis reopened.

We found that the duration of the second SSW significantly increased as the volume of the bolus increased. Although Cichero reported that the swallowing sound duration decreased and the intensity remained unchanged as the bolus volume increased [21], Boiron and Youmans reported that the swallowing sound duration increased as the bolus volume increased [22,23]. Furthermore, Hammoudi reported that the duration of the second component of the swallowing sound increased as the bolus volume [24]. The second SSW in the present study is likely identical to the swallowing sound waveform analyzed in previous studies. During videofluoroscopy of the second SSW, the bolus was transferred from the pharynx to the esophagus through the esophageal orifice. The prolongation of the second SSW with the increased bolus volume likely reflects the increased transit time from the pharynx to the esophagus through the esophageal orifice. The incremental increase in the peak intensity ratio between the second and third SSWs with the increased bolus volume may be caused by the increased muscle effort required to swallow the greater volume.

The head and cervical postures will influence morphological changes in the bolus pathway during swallowing and consequently, may change the acoustic characteristics of the swallowing sound [25]. The duration of the first SSW was significantly shorter in the chin-down position than in the normal chin position; conversely, the mean duration of the second SSW was significantly longer in the chin-down position than in the normal chin position. No significant difference was observed between the two positions in the duration of the third SSW, and the peak intensity ratio did not differ significantly according to the cervical posture in any of the SSW periods. Logemann reported that the chin down posture caused the anterior pharyngeal structures to shift posteriorly, narrowing the laryngeal entrance and decreasing the distance from the epiglottis to the pharyngeal wall and laryngeal entrance [26]. This posterior shift during the chin down position helps protect the airway. Karaho reported that the chin-down maneuver decreases the distance between the tongue base and the posterior pharyngeal wall, the duration of anterior hyoid movement, and the duration of upper esophageal sphincter opening [27]. Therefore, we suspect that the chin-down position decreased the distance between the tongue base and posterior pharyngeal wall, and facilitated bolus transportation from the oral cavity to the pharynx in the present study. Ultimately, the duration of the first SSW would be shortened during the chin down position. Presumably, the prolonged second SSW during the chin down position was caused by the shortened duration of the first SSW, and the increased time and space for bolus transportation reflected the morphological change.

The videofluoroscopy measurement in experiment 3 involved only three male participants. Although Dantas reported oropharyngeal transit of food during swallowing in women was longer than that in men [28], no significant difference of any measurements was found between genders in the present experiment 1. This could be a major limitation of the present study.

## Conclusions

In conclusion, acoustic analysis of swallowing sounds and videofluoroscopy suggests that the swallowing sound could be divided into three periods associated with each process the swallowing movement: an oral phase comprising posterior movement of the tongue and hyoid bone; a pharyngeal phase comprising laryngeal movement, hyoid bone elevation, epiglottis closure, and the bolus passage to the esophagus orifice; and the repositioning phase comprising repositioning of the hyoid bone and larynx, and reopening of the epiglottis.

However, the variations of swallowing sound values were relatively large in inter-subjects, but stable in intra-subject as shown in Table 1, and it may show the low reliability of swallowing sound for dysphagia examination. The examination may be improved by associating the wave structure with physiological events during the examination. Further research conducted in dysphagia patients is required to validate the use of cervical auscultation for dysphagia examination.

## Supporting Information

S1 Appendix Acoustic data including duration and peak intensity ratio in Experiment 1 and 2. (XLSX)
